# The preferential accumulation of heavy metals in different tissues following frequent respiratory exposure to PM_2.5_ in rats

**DOI:** 10.1038/srep16936

**Published:** 2015-11-19

**Authors:** Qingzhao Li, Huibin Liu, Mohamed Alattar, Shoufang Jiang, Jing Han, Yujiao Ma, Chunyang Jiang

**Affiliations:** 1 School of Public Health, North China University of Science and Technology, Jianshe Road 57, Tangshan 063001, Hebei, People’s Republic of China; 2 Office of Clinical Drug Trial Institution, The Affiliated Tumor Hospital of Xinjiang Medical University, Urumqi, 830011, Xinjiang, People’s Republic of China; 3 Department of Cardiothoracic surgery, Zagazig University hospital, faculty of medicine, Zagazig University, Sharkia 44519, Egypt; 4 Department of Thoracic Surgery, Tianjin Union Medicine Centre, 190 Jieyuan Road, Hongqiao District, Tianjin 300121, People’s Republic of China

## Abstract

This study aimed to explore the pattern of accumulation of some of main heavy metals in blood and various organs of rats after exposed to the atmospheric fine particulate matter (PM_2.5_). Rats were randomly divided into control and three treatment groups (tracheal perfusion with 10 mg/kg, 20 mg/kg and 40 mg/kg of PM_2.5_ suspension liquid, respectively). Whole blood and the lung, liver, kidney, and cerebral cortex were harvested after rats were treated and sacrificed. The used heavy metals were detected using inductively coupled plasma-mass spectrometry (ICP-MS) instrument. As results, Lead was increased in the liver, lung and cerebral cortex and the level of manganese was significantly elevated in the liver and cerebral cortex in PM_2.5_ treated rats. Besides, arsenic was prominently enriched both in cerebral cortex and in blood, and so did the aluminum in the cerebral cortex and the copper in the liver. However, cadmium, chromium and nickel have shown no difference between the control group and the three PM_2.5_ treated groups. Following the exposure of PM_2.5_, different heavy metals are preferentially accumulated in different body tissues.

Global air pollution became more serious in the recent years and posed public health and safety concerns. Atmospheric particulate matter (PM) is a kind of solid or liquid complex compounds suspended in the atmosphere and a main source of atmospheric pollution. PM, especially fine particulate matter (PM_2.5_), which has a diameter of no more than 2.5 μm, causes serious harm to human health because of its complicated composition, strong adsorption and rising levels in tandem with rapid industrial development^1^. It was recognized as the most representative of the atmospheric pollutants. Its monitoring attracts more and more attention worldwide as it aggravates many health problems on prolonged exposure^2^,^3^,^4^.

Because PM_2.5_ has a long residence time of several days to several weeks in atmosphere, it can travel hundreds to thousands of kilometers. The fine particles in ambient air have been reported to be associated with many health problems including respiratory symptoms, asthma exacerbations, and decrements in lung function^5^,^6^. Except for certain insoluble inorganic substances and hydrophobic substances, PM_2.5_ with water soluble and hygroscopic characteristics could be bio-available^7^,^8^. For its large surface area and strong adsorption capacity, PM_2.5_ can adsorb, combine and transport polycyclic aromatic hydrocarbon (PAH), polychlorinated biphenyls (PCB), heavy metals, bacteria, viruses and other toxic substances and potential carcinogens^9^,^10^,^11^. For insoluble components of PM_2.5_, once these particulates had been inhaled into the low respiratory tract, they could not only cause inflammatory damage to lung tissues and change the state of relaxation and contraction of blood vessels, but also could diffuse through the alveolar wall into the blood circulation and cause a widespread harm to the body^12^,^13^,^14^,^15^.

Studies confirmed that PM_2.5_ with mutagenicity could increase mortality, damage the immune system, as well as cause abnormalities of the nervous system and other serious harm^16^,^17^. PM_2.5_ contains high concentrations of toxic trace metals, such as chromium (Cr), cadmium (Cd), titanium (Ti), manganese (Mn), nickel (Ni), lead (Pb), arsenic (As), zinc (Zn), etc.^18^,^19^. These toxic heavy metals incorporated with atmospheric PM_2.5_ may enter the body through inhalation and have been suggested as causative agents associated with adverse respiratory health effects. Additionally, they can gather in different parts of the body. Heavy metal is not easily biodegradable, and prone to accumulate to hundreds of thousands of times through the food chain under the action of biological amplification enrichment. Synergism or antagonism would occur between all kinds of heavy metal elements in different organisms. A heavy metal element can affect the absorption of another or change its distribution in the body. Studies have shown that Pb, Cd, Cr and Ni in low concentrations from PM_2.5_ *in vivo* or *in vitro* can exhibit genetic toxicity through producing primary DNA or chromosomal damage^20^. However, researches about intracorporal metabolic distribution of PM_2.5_ in the major organs are still insufficient.

This study aims at analyzing and comparing the main heavy metals contents of PM_2.5_ including Pb, aluminum (Al), Mn, copper (Cu), As, Cd, Cr and Ni elements in the blood, lung, liver, kidney, and cerebral cortex of rats after establishment of a rat model which is chronically infected with PM_2.5._ Eventually, these experimental data can provide scientific evaluation for studying the mechanisms of toxicity induced by atmospheric PM_2.5_.

## Materials and Methods

### Reagents and instruments

Normal saline (NS) was obtained from Shandong kangning pharmaceutical Co., Ltd (Shandong, China); Absolute ethyl alcohol was gained from Samtec Tianjin Chemical Reagent Co., Ltd. (Tianjin, China); Diethyl ether and Nitrate (with an excellent level of purity) were purchased from Beijing Chemical Works (Beijing, China); Perchloric acid was purchased from Tianjin zhengcheng chemical products Co., Ltd (Tianjin, China).

TH-150D II PM Sampler was purchased from Wuhan Tianhong Instruments Co., Ltd. (Wuhan, China); Agilent 7500a inductively coupled plasma-mass spectrometry (ICP-MS) was produced from Thermo Scientific Co., Ltd. (Agilent, Santa Clara, USA); Aquaplore ultra-pure water system AWL-2002-Μ was gained from Shanghai bettersize Co., Ltd. (Shanghai, China); ETHOSA Microwave Digestion System (MILESTONE Co., Ltd, USA).

### The preparation of mixed PM_2.5_ suspension

The atmospheric PM_2.5_ sample was provided by the environmental monitoring center of Tangshan city and the sampling location was at the roof of that center. The sample was collected from December 15, 2013 to February 15, 2014 during the winter season of the city. About 100 m^3^ sample of air was collected over 24 hours per day each time.

The membrane filter carrying PM_2.5_ was put into the ultra-pure water and the particles were eluted by ultrasonic oscillator. After 30 min of oscillation, the supernatant fluid was filtered by 5-layer sterile gauze. The obtained liquids were dried to get PM_2.5_ particles. Control membrane filter was procedurally treated with ultrasonic oscillation in NS as above mentioned and the liquid was utilized in control animals.

PM_2.5_ particles were weighted and dissolved in NS to make a 4 mg/ml stock solution and the liquid was preserved at 4 °C. Before using, the suspensions were preceded by 30 min ultrasonic oscillation to scatter the particles and then sterilized by autoclaving.

### Animal treatment with PM_2.5_

The 48 adult specific-pathogen-free (SPF) Sprague-Dawley male rats weighting 200–220 g were purchased from the Institute of Hygiene and Environmental Medicine, Academy of Military Medicine (the license number was SCXK- (Army) 2009-003 and the certificate of conformity number was 0001596). The rats were randomly divided into four groups, namely the control group and three treatment groups. They were free feeding and drinking for one week. After ether drugged, each rat in three exposed groups was administrated with PM_2.5_ working solution (10 ml/kg·body weight) by tracheal perfusion. The exposed dosages used in this study for three groups were 10 mg/kg, 20 mg/kg and 40 mg/kg, respectively. Each working solution was freshly prepared by diluting stock solution with NS. For control group, each rat was treated by the same method with NS (10 ml/kg·body weight) which was processed by the oscillation of the control membrane filter. These experimental rats were treated once a week for up to 12 times. All the experimental protocols were approved by ethics committee of North China University of Science and Technology, Tangshan, Hebei province, China. The methods were carried out in accordance with the approved guidelines.

After finishing the last adminstration, all rats were sacrificed 5 days later. The whole bloods were gathered and the lung, liver, kidney as well as cerebral cortex were removed. All biological samples were immediately stored at −20 °C. 0.1 g of the specimens was respectively put into a small beaker and then digested with 4 ml of mixed concentrated acid (perchloric acid: nitric acid as 1: 4) for 12 h. After that, the beakers were placed on one electric hot plate until white crystal appeared at the bottom of the containers. The capacity was fixed to 5 ml by adding dilute nitric acid (1%) after cooling. Eventually, the contents of heavy metal elements in these samples were determined by using ICP-MS instrument.

### The detection of the heavy metal elements

Agilent 7500a ICP-MS was employed to measure the contents of eight kinds of heavy metal elements in these samples. The working conditions and the instrument parameters were listed in table 1. Agilent Calibration Verification Standard solutions were diluted with 1% HNO3 to obtain the standard liquids (STD1). For each heavy metal element, STD1 was diluted into 6 different concentrations by multiple. For STD1, the minimum concentration was 0 ug/L for all these heavy metal elements and the maximum concentrations were 200 ug/L for Al, Pb, Cu, Mn, As, Cr and 20 ug/L for Cd and Ni, respectively. The internal standard elements solution (ISTD, 1 ug/ml) was made by dilution of 10 μg/ml Li6, Sc, Ge, Y, In, Tb as well as Bi and 1% HNO3 was used as the blank (STD0). The ICP-MS was equipped with an autosampler and an Integrated Sample Introduction System with Discrete Sampler (ISIS-DS). A Micromist glass concentric nebulizer (Glass Expansion, MA, USA), quartz torch with a 2.5 mm diameter injector and Shield Torch Technology (Agilent Technologies, CA, USA) were used in the detection.

### Statistic analysis

All data were analyzed using One-way univariate analysis of variance (ANOVA) followed by Tukey (equal variances assumed or homogeneity of variance after the variable transformation) or Dunnett’s T3 (equal variances not assumed after the variable transformation justification) for Post Hoc test between groups using Statistical Package for Social Sciences software (SPSS version 16.0, Chicago, IL, USA). The results were represented as mean ± SD. All tests were two sided, *P* < 0.05 was considered statistically significant.

## Results

### The contents of heavy metal elements in PM_2.5_ particles

**Figure 1:**
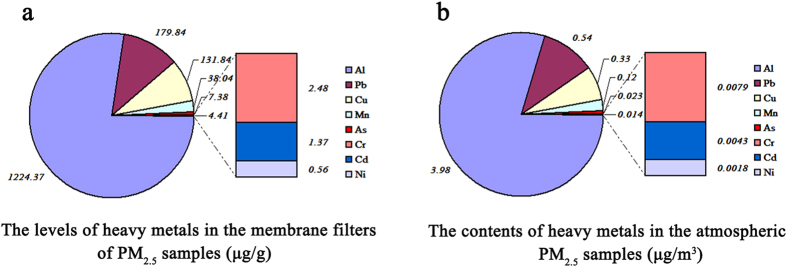
The measurements of heavy metal elements in atmospheric PM_2.5_ particles by ICP-MS during winter in tangshan city. The levels of heavy metals in the membrane filters of PM_2.5_ samples are shown in (**a**). The contents of heavy metals in the atmospheric PM_2.5_ samples are shown in (**b**). The eight kinds of heavy metal elements were displayed in order as Al > Pb > Cu > Mn > As > Cr > Cd > Ni.

After collection, the membrane filters carrying PM_2.5_ particles were processed and their heavy metal elements were detected by ICP-MS. The results are shown in Fig. 1(a). The contents of eight kinds of heavy metal elements in the atmospheric PM_2.5_ samples were ranked from the highest to the lowest level as follows: Al, Pb, Cu, Mn, As, Cr, Cd and Ni. The contents of the first four kinds of metals were higher than 38.0 μg/g. The component analysis showed that airborne concentrations of Al and Pb were the highest (3.98 μg/m^3^ and 0.54 μg/m^3^, respectively) among the eight metals of this city’s atmospheric particulate matter in winter, and the contents of rest were followed by Cu (0.33 μg/m^3^), Mn (0.12 μg/m^3^), As (0.023 μg/m^3^), Cr (0.0079 μg/m^3^), Cd (0.0043 μg/m^3^) and Ni (0.0018 μg/m^3^). The results are shown in Fig. 1(b).

### Heavy metal contents in blood and visceral organs of rats

The eight kinds of heavy metal elements including Al, Pb, Cu, Mn, As, Cr, Cd and Ni in the whole bloods and the liver, lung, cerebral cortex and kidney of rats were detected by ICP-MS.

**Figure 2:**
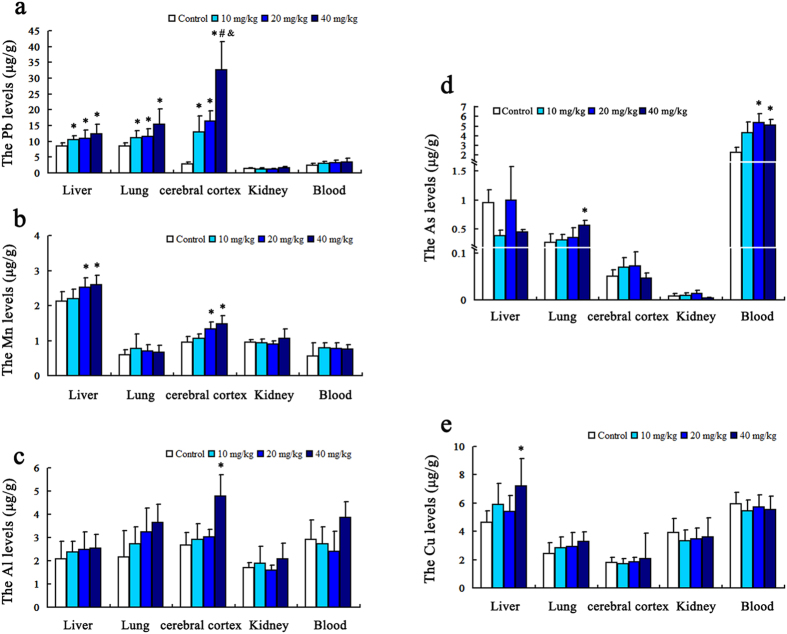
The levels of heavy metal elements in the whole blood, liver, lung, cerebral cortex and kidney of rats. Five heavy metals, including Al, Pb, Cu, Mn and As, showed significantly higher levels in groups treated with PM_2.5_ than control group. The results were shown in (**a**–**e**), representing Pb, Mn, Al, As and Cu respectively. ^*^P < 0.05 = significant as compared to the control; ^#^*P* < 0.05 = significant as compared to the 10 mg/kg group; ^&^*P* < 0.05 = significant as compared to the 20 mg/kg group (n = 12).

As shown in Fig. 2(a), the visceral lead contents in rats treated with PM_2.5_ (10 mg/kg, 20 mg/kg and 40 mg/kg) were significantly higher than the control group; the differences were statistically significant (*P* < 0.05) (F = 3.54, *P* = 0.033; F = 7.09, *P* = 0.002 and F = 5.10, *P* = 0.011, respectively for 40 mg/kg groups, n = 12). Furthermore, lead concentration in the cerebral cortex is creeping upward with the increasing dose and it indicated a remarkable dose-effect relationship.

The results of manganese are shown in Fig. 2(b). Compared with control group, manganese contents of rat’s liver and cerebral cortex in the middle and high dose groups were obviously increased (*P* < 0.05) (F = 3.82, *P* = 0.026 and F = 7.30, *P* = 0.003 for liver; F = 4.78, *P* = 0.013 and F = 9.22, *P* = 0.002 for cerebral cortex, respectively, n = 12).

As for aluminum, its content was significantly higher than those of the control group only in rats’ cerebral cortex of the high dose group (*P* < 0.05) (F = 3.616, *P* = 0.04), (n = 12), as displayed in Fig. 2(c).

The comparison of arsenic contents in different groups is shown in Fig. 2(d). Compared with control group, the contents in the lung of the high dose group (F = 4.14, *P* = 0.021) and in the total blood of both the middle and the high dose groups (F = 6.589, *P* = 0.003; F = 10.649, *P* = 0. 001) were significantly greater (*P* < 0.05), (n = 12). The copper content in the liver of 40 mg/kg group was higher than the control group and the difference was significant (F = 3.475, *P* = 0.035). The results were shown in Fig. 2(e).

**Figure 3:**
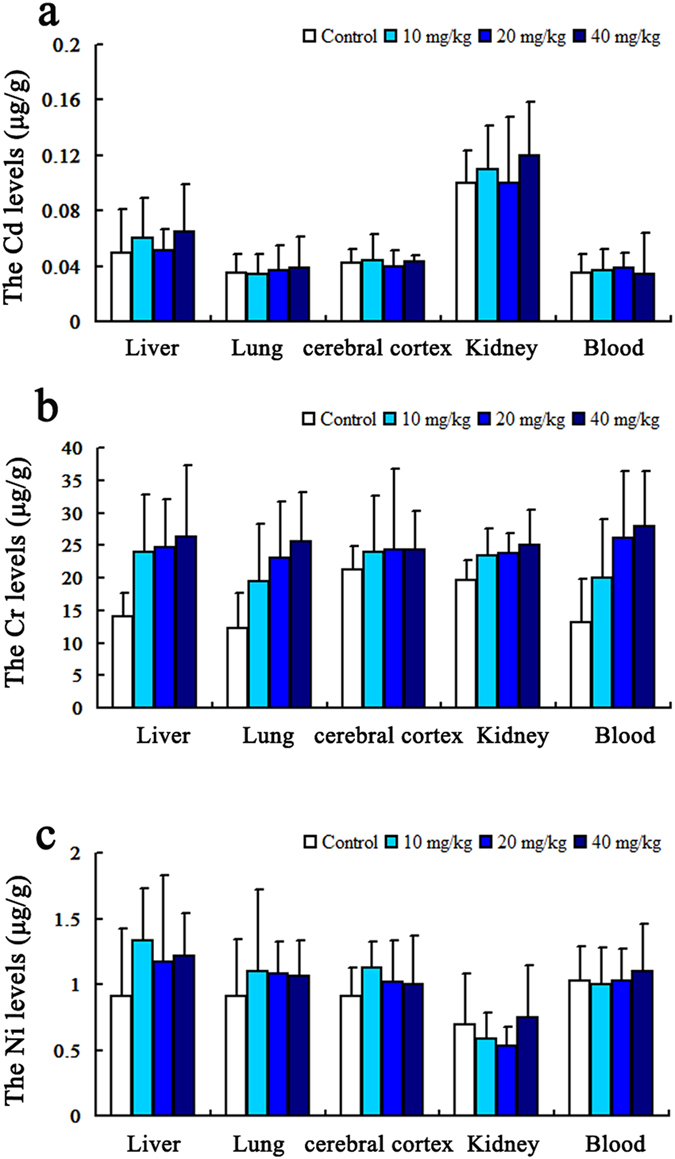
The levels of heavy metal elements in the whole blood, liver, lung, cerebral cortex and kidney of rats. Three heavy metals, including Cd, Cr, and Ni, showed no significant difference between the control group and the PM_2.5_ treated groups. The results were shown in (**a**–**c**). (*P* > 0.05, respectively) (n = 12).

The results of cadmium, chromium and nickel elements were shown in Fig. 3(a–c). There were no difference between the control groups and the PM_2.5_ treated groups regarding the concentrations of these elements in rat’s blood and viscera.

## Discussion

Nowadays, the particulate matter is a main factor affecting global air quality and a primary pollutant for most of the industrial cities. In this study, PM_2.5_ was collected from Tangshan city which is a heavy industrial port in the north of China with many coal-fired power station, coke-oven plants and iron and steel plants being there. Besides Tangshan, Beijing and Tianjin are also the main areas of atmospheric particulate matter pollution in northern China^21^.

The analysis of heavy metal components showed that the fine particles PM_2.5_ in this city primarily consist of aluminum, lead, copper and manganese. In the field of environmental pollution, heavy metal mainly refers to those metal or metalloid elements with obvious biotoxicity such as mercury, cadmium, lead, chromium, copper, cobalt, nickel, tin, arsenic, aluminum, etc. Such pollutants are not easily be degraded by microorganism and may even undergo bioamplification^18^. Although some other heavy metals are essential elements and a small amount of them show the health benefits such as chromium and manganese, yet their excessive intake can cause damage^22^. Some heavy metals such as lead and arsenic are well known to be toxic to human body. PM_2.5_ is an important carrier of heavy metals, and as an atmospheric pollutant, it has potentially serious health hazard to the residents of the contaminated areas^16^.

Lead as a heavy metal element can pass the blood-brain barrier (BBB), accumulate in brain and eventually cause damage to the central nervous system^23^. The present study showed that lead was more prominent in the liver, brain and lung of rats when exposed to PM_2.5_ than the control group. This may be because PM_2.5_ in the systemic circulation has access to each organ system of the body and they are selectively accumulated in some organs. The liver is the main detoxification organ and accordingly it may have high content of the accumulated lead. Brain may be another important target organ for lead accumulation due to slow excretion^24^. Brain tissues are relatively sensitive to microenvironment changes. Therefore, even trace amounts of lead can also accumulate in brain tissue and induce neurotoxicity. In this study, with the increase of infected dose, the elevated lead levels in rats’ cortex are obviously detected, presenting significant dose-effect relationship. Kidney is the main excretory organ and it has faster metabolic rate than other organs^25^,^26^, so there were no obvious difference in lead levels between the experimental and control groups.

Manganese is one of essential trace elements in different metabolic processes, and as a co-factor of oxidative phosphorylation, it is needed in the enzyme system for catalysing this sequence of oxidative reactions^27^. Manganese can enter the systemic circulation before being uptaken by mitochondria rich cells in liver, brain, and hair^28^. High levels of manganese can induce toxic effect on multiple organs so it adversely affects the functions of the liver, cardiovascular, reproductive, immune system and central nervous system^29^. A study has shown that manganese can pass through the BBB of newborn rat and induce damaging effect on hippocampal development, which finally results in neurobehavioral changes of newborn^30^.

Due to slow excretion in the brain, excessive accumulation of manganese is the reason that brain is the most affected organ of manganese toxicity^31^. According to our results, both middle and high dose exposure can lead to selective accumulation of the excessive manganese in the brain and liver, which may induce target organs damage.

The main toxic effect of aluminum is exerted on the nervous system. Aluminum can combine with the phospholipids by complexation and affect the function of nerve cell membrane. Aluminum can also bind the phosphate group in the nuclear chromatin of neurons and disturb DNA transcription and replication to result in abnormal metabolism and protein synthesis^32^. In addition, it can interfere with cellular energy status and bring about changes in cholinergic neurotransmitter and destruction of BBB function to cause dementia or other degenerative diseases^33^,^34^. Related studies have shown that long-term exposure of aluminum increases the susceptibility to Alzheimer’s disease^35^. In this study, high dose exposure to PM_2.5_ significantly increased the content of aluminum in cerebral cortex, which confirmed that aluminum can pass the BBB and tend to accumulate in the brain.

The symptoms of arsenicism may appear very soon or may appear after more than ten years or even decades^36^. It primarily depends on the nature of exposure including the amount and duration of intake of arsenic compounds and the general individual health condition. Unbound arsenic exerts its toxicity by generating reactive oxygen intermediates during their redox cycling and metabolic activation processes that may cause lipid peroxidation and DNA damage. Moreover, it can bind thiol or sulfhydryl groups in tissue proteins of the liver, lung, kidney, spleen, gastrointestinal mucosa, and keratin-rich tissues (skin, hair, and nails)^37^. Chronic arsenic exposure may be associated with the higher probability of lung cancer occurrence^38^. This study has found that after exposure to PM_2.5_, the accumulation of arsenic in blood and lung were obviously increased in a dose dependent manner. Thus it can be understood that hematopoietic dysfunction and an increase in the risk of lung cancer are related to the effect of chronic arsenic exposure.

Just like other essential trace elements, excessive intake of copper can also cause toxic reaction. Copper is mainly concentrated in the liver and once the amount exceeds the ability of liver detoxification, it would be released into the blood^39^,^40^. Chronic copper poisoning can cause hepatomegaly and abnormal liver function^41^. In addition, chronic copper poisoning can lead to lung fibrosis and nervous system disorders including poor memory and attention, instability, multiple neuritis^42^,^43^. Selective hepatic lodging of copper was proved by this study so it can be inferred that long-term exposure to PM_2.5_ would probably lead to liver damage in the first instance.

Although chromium is one of the essential elements, long-term exposure to chromium compounds can lead to lung cancer and hepatocellular carcinoma^44^. Respiratory tract is the main entry port of cadmium resulting inhalation toxicity, and likewise, it may induce acute liver and kidney damage as well as chronic damage of many organs and systems^45^. Nickel represents a good example of a metal whose use is increasing in modern technologies. Among the known health related effects of nickel are skin allergies, lung fibrosis, various degrees of kidney injury, cardiovascular system deterioration and stimulation of neoplastic transformation. Nevertheless, the mechanisms of these effects remain not well known^46^. The results of our study showed that there was no difference of chromium, cadmium and cickel between control group and those treated with different concentrations of PM_2.5_ in the whole blood and mainly organs of rats. This may be correlated to the low levels of these elements in PM_2.5_ samples.

Collectively, PM_2.5_ is a complex mixture of a variety of constituents. After sedimentation in the lung, the heavy metals in PM_2.5_ particles can easily get into the circulatory system and then accumulate in the target organs such as liver, brain and kidney to cause their dysfunction. They have not only teratogenic, carcinogenic and mutagenic effects but also a huge potential damage to their target organs.

The present studies about *in-vivo* metabonomics research of important PM_2.5_ constituents are very limited and there is a need to strengthen the basic research and the epidemiological investigations. This study showed that heavy metals such as lead, manganese, aluminum, and copper carried by PM_2.5_ can enter the circulation after respiratory exposure and selectively accumulate in the target organs including blood, brain, liver and lungs. This study of heavy metals distribution and metabolism in rat body highlights the potential harm of the atmospheric PM_2.5_ to various organs of rat providing scientific basis for further studies on the health hazards of the atmospheric PM_2.5_.

## Additional Information

**How to cite this article**: Li, Q. *et al.* The preferential accumulation of heavy metals in different tissues following frequent respiratory exposure to PM_2.5_ in rats. *Sci. Rep.* **5**, 16936; doi: 10.1038/srep16936 (2015).

## Figures and Tables

**Table 1:** Operating parameters for 7500a ICP-MS instrument.

Parameters	Setting
Flow rate of carrier gas (L/min)	1.14
Sampling depth (mm)	5.2
Radio-frequency power (W)	1480
Spray chamber temp (°C)	2
Sample cone	Nickel Skimmer
Sampling pattern	Quantitative
Scanning mode	Jump peak
Times of repetition	3
